# Extending the depth-of-field of imaging systems with a scattering diffuser

**DOI:** 10.1038/s41598-019-43593-w

**Published:** 2019-05-09

**Authors:** Meihua Liao, Dajiang Lu, Giancarlo Pedrini, Wolfgang Osten, Guohai Situ, Wenqi He, Xiang Peng

**Affiliations:** 1 College of Optoelectronics Engineering, Key Laboratory of Optoelectronic Devices and Systems of Ministry of Education and Guangdong Province, Shenzhen University, Shenzhen, 518060 China; 2 Institut fuer Technische Optik, University of Stuttgart, Pfaffenwaldring 9, 70569 Stuttgart, Germany; 3 Shanghai Institute of Optics and Fine Mechanics, Chinese Academy of Sciences, Shanghai, 201800 China

**Keywords:** Imaging and sensing, Micro-optics

## Abstract

Large depth of field (DOF) is a longstanding goal in optical imaging field. In this paper we presented a simple but efficient method to extend the DOF of a diffraction-limited imaging system using a thin scattering diffuser. The DOF characteristic of the imaging system with random phase modulation was analyzed based on the analytical model of ambiguity function as a polar display of the optical transfer function (OTF). The results of numerical simulation showed that more high-frequency components existed in the defocused OTF curve when the exit pupil of the imaging system exhibited a random phase modulation. It proved the important role of the scattering diffuser in extending the DOF of imaging systems. For the reconstruction, a stack of point spread functions (PSFs) corresponding to different axial locations within a measurement range were superimposed to construct the stacked PSF. Then the large DOF image was recovered from a speckle pattern by deconvolution. In this proof-of-concept, we experimentally demonstrated the single-shot imaging with larger DOF using a thin glass scattering diffuser in both a single-lens imaging system and a microscopic imaging system.

## Introduction

The depth-of-field (DOF) of an imaging system refers to the range in the scene that will appear in focus. The parts of the image that lie outside the DOF tend to be blurred and less sharp. Optical imaging with a large DOF is a longstanding goal with important applications in varied fields^1–4^. In general, the increase of DOF are associated with a reduction of the numerical aperture or the use of an optical power absorbing apodizer^5^. However, it leads to degrading of the optical power at the image plane and decrease of resolution. To overcome these limitations, a variety of techniques have been proposed over the past years. One representative is image fusion where several images of the same scene are acquired while different parts of the object are focused on^3,6^. But it is time-consuming and multiple-exposure is not applicable to dynamic scenes. Another promising technique is wavefront coding, in which the incoherent optical system is modified using a cubic-phase mask (or other special phase mask). As a result, the point spread function (PSF) is not sensitive to defocusing^7–14^. However, the phase masks need to be specially designed and fabricated, which is much difficult as the phase mask has spatially varied thickness and is combined with non-rotationally symmetric aspheric optical elements. Furthermore, the optical recording process requires an accurate alignment of these special phase masks since the decenter and tilt may degrade the imaging performance^15^.

Imaging of objects within or hidden behind diffusing media is challenging due to severe degradation in image quality caused by random scattered light. Continuous efforts have been invested to find a solution to seeing through a scattering medium^16–19^. On the other hand, a scattering medium has been considered as a helpful optical element^20–27^ for imaging instead of an undesirable obstacle in recent years. As reported in literature, scattering improved the sharpness of the focus via spatial wavefront shaping^20^; a randomly scattering high-index medium successfully enhanced the resolution of wide-field fluorescence microscopy^21^; the scattering matrix measurement realized the reference-free holographic imaging^22^; the spectral decorrelation effect in scattering media resulted in single-shot multispectral imaging^23^; the use of scattering media could also achieve 3D imaging^24,25^ and lensless microscopy^26^; DiffuserCam achieved lensless 3D imaging with a well-designed architecture and compress sensing algorithm^27^. Among these works mentioned above, few showed the ability to extend DOF. Speckle-correlation-based imaging experimentally demonstrated the property of infinite DOF^28^ but no axial sectioning capability. Recently, some contributions have been made to depth-resolved and DOF-extended imaging through scattering media, such as phase space measurement^29^, PSF manipulation^30^, light field estimation^31^ and coherence gating^32^. Nevertheless, their practical application is limited by complex experimental setup and tedious calculation.

Here, we presented a simple but efficient technique to extend the DOF of an incoherent diffraction-limited imaging system using a thin scattering diffuser such as a common ground glass. The DOF characteristic of the imaging system with scattering medium was analyzed based on the analytical model of the ambiguity function as a polar display of the optical transfer function (OTF). It showed more high-frequency components were collected by the scattering medium, which were normally lost with defocusing in traditional lens-based imaging systems. Experiments demonstrated large DOF images could be reconstructed using the PSF stack calculated from a sequence of PSFs.

## Principle and Method

### Analysis on the DOF characteristic of the imaging system with a scattering diffuser

For the sake of simplicity and without loss of generality, we consider an example of a single-lens imaging system. Assume a thin scattering diffuser is a random pure phase function, in normalized coordinates, that is

1 $$P(x)=\{\begin{array}{c}exp[j2\pi R(x)],\\ 0\end{array}\begin{array}{c}|x|\le 1\\ otherwise\end{array}$$

where *x* is the normalized pupil coordinate, *R*(*x*) is a random distribution in the range between [0,1]. Thus the generalized pupil function, as a function of misfocus, can be expressed as

2 $$Q(x)=P(x)exp[jk{W}_{20}{x}^{2}]$$

where *k* is the wave number and *W*_20_ denotes the misfocus aberration constant. According to Hopkins criterion^7^, the one-dimensional defocused optical transfer function (OTF) that takes misfocusing into account is:

3 $$H(u;{W}_{20})=\int Q(x+\frac{\mu }{2}){Q}^{\ast }(x-\frac{\mu }{2})dx=\int P(x+\frac{\mu }{2}){P}^{\ast }(x-\frac{\mu }{2})exp[j2\pi (\frac{2{W}_{20}}{\lambda })x\mu ]dx$$

where *λ* is the wavelength of the illuminated light, *μ* denotes spatial frequency coordinate. On the other hand, the ambiguity function (AF) in the phase space is defined as:

4 $$A(u;y)=\int P(x+\frac{\mu }{2}){P}^{\ast }(x-\frac{\mu }{2})exp[j2\pi xy]dx$$

where *y* denotes the normalized coordinate. Based on comparison of Eqs (3) and (4), the two-dimensional AF can be regarded as a polar representation of the one-dimensional OTF of a rectangular separable incoherent optical system with the focus error as variable^7,8^, that is:

5 $$H(\mu ;{W}_{20})=A(\mu ;\frac{2{W}_{20}}{\lambda }\mu )$$

**Figure 1:**
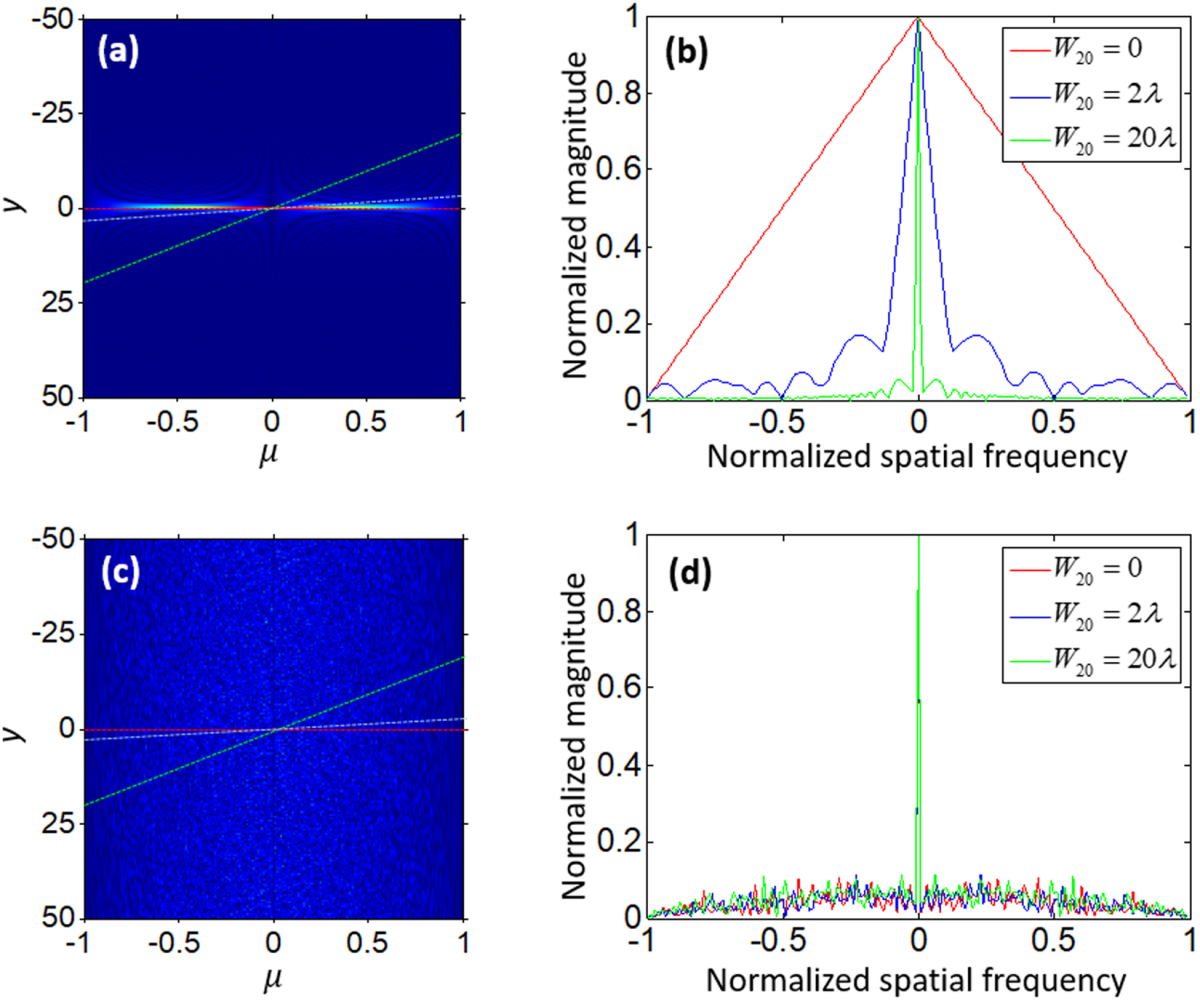
The defocused OTFs characteristics by numerical simulation. (**a**) AF of a rectangular aperture function. (**b**) The defocused OTF curves of the standard optical imaging system. (**c**) AF of a rectangular aperture function with a random phase function. (**d**) The defocused OTF curves of the scattering diffuser system.

In such way, AF could be used as an analytical tool that allows us to observe the OTF for all values of misfocus simultaneously. Numerical simulations were carried out to explore OTF’s statistical properties. An incoherent optical system with a one-dimensional rectangular aperture was taken into account. The corresponding two-dimensional AF of the exit pupil function is shown in Fig. 1(a). From Eq. (5), the defocused OTFs of the standard optical system with three different levels of defocusing (*W*_20_ = 0, *W*_20_ = 2*λ* and *W*_20_ = 20*λ*) were calculated. The results are shown in Fig. 1(b), where the horizontal and vertical axis denote the normalized spatial frequency and magnitude of the OTF, respectively; red, blue and green curves present the magnitude of OTFs without defocus (*W*_20_ = 0), with slight defocus (*W*_20_ = 2*λ*) and with severe defocus (*W*_20_ = 20*λ*), respectively. The high-frequency components became increasingly smaller with the increase of the defocusing. In the proposed imaging system, a scattering diffuser with random phase was inserted as the exit pupil function and its two-dimensional AF is shown in Fig. 1(c). Similarly, we plotted the defocused OTFs for the same amounts of defocus as shown in Fig. 1(d). Compare the green line in Fig. 1(b,d), a random character but more high-frequency components were found in the severe defocused OTF curves. It indicated that the scattering medium contributed to collection of more high-frequency components that were lost with defocusing in traditional imaging systems. Therefore, a thin scattering diffuser could be consider as a helpful optical element for extending the DOF of a standard lens-based imaging system.

### Extending the DOF of an imaging system with a thin scattering diffuser

This section describes how to extend the DOF of a diffraction-limited imaging system using a thin scattering diffuser. In a standard lens-based imaging system, a thin scattering diffuser as an element was placed at the exit pupil. When light illuminating the object, passes through the lens, the scattering diffuser, and finally forms a speckle pattern at the camera plane. Under incoherent illumination, the light from nearby points within the range of “memory effect” on the object is scattered by the thin diffuser, resulting in highly correlated but shifted PSFs that are formed on the output plane^33–35^. Then the recorded speckle pattern is simply a superposition of these identical shifted PSFs. Therefore, the system with a thin scattering diffuser is approximately regarded as an incoherent imaging system, the speckle pattern intensity *I* is nearly equal to the convolution of the object *O* with the intensity PSF *S*, i.e. *I* = *O*   * *S*. If objects are located at *n* different axial positions *z*, the recorded speckle pattern can be written as

6 $$I={\sum }_{z=1}^{n}{I}_{z}={\sum }_{z=1}^{n}({O}_{z}\ast {S}_{z})={\sum }_{z=1}^{n}{O}_{z}\,\ast \,{\sum }_{z=1}^{n}{S}_{z}+C$$

where the symbol “*” and “∑” are the convolution and matrix addition operation, *C* denotes the sum of all the convolution cross terms, which should be approximately flat when *n* is big enough due to the cross terms are different for the different PSFs. When a set of PSFs (PSF stack) corresponding the *n* axial positions in the whole measurement range are recorded in advance (it is reasonable because the scattering diffuser is employed intentionally as an optical element into the imaging system), all the object *O* located at different axial planes can be recovered from its speckle pattern *I* with the overlays of all the PSFs by deconvolution^35^:

7 $$O\approx { {\mathcal F} }^{-1}\{\frac{ {\mathcal F} \{I\}}{ {\mathcal F} \{\sum {S}_{z}\}}\}$$

where ${\mathcal F}$ and ${ {\mathcal F} }^{-1}$ denote 2D Fourier transform and 2D inverse Fourier transform, respectively. Moreover, as the axial positions of the whole calibrated PSF stack are known in advance, that allows to retrieve each layer of object with the corresponding depth information.

## Experimental Verification

**Figure 2:**
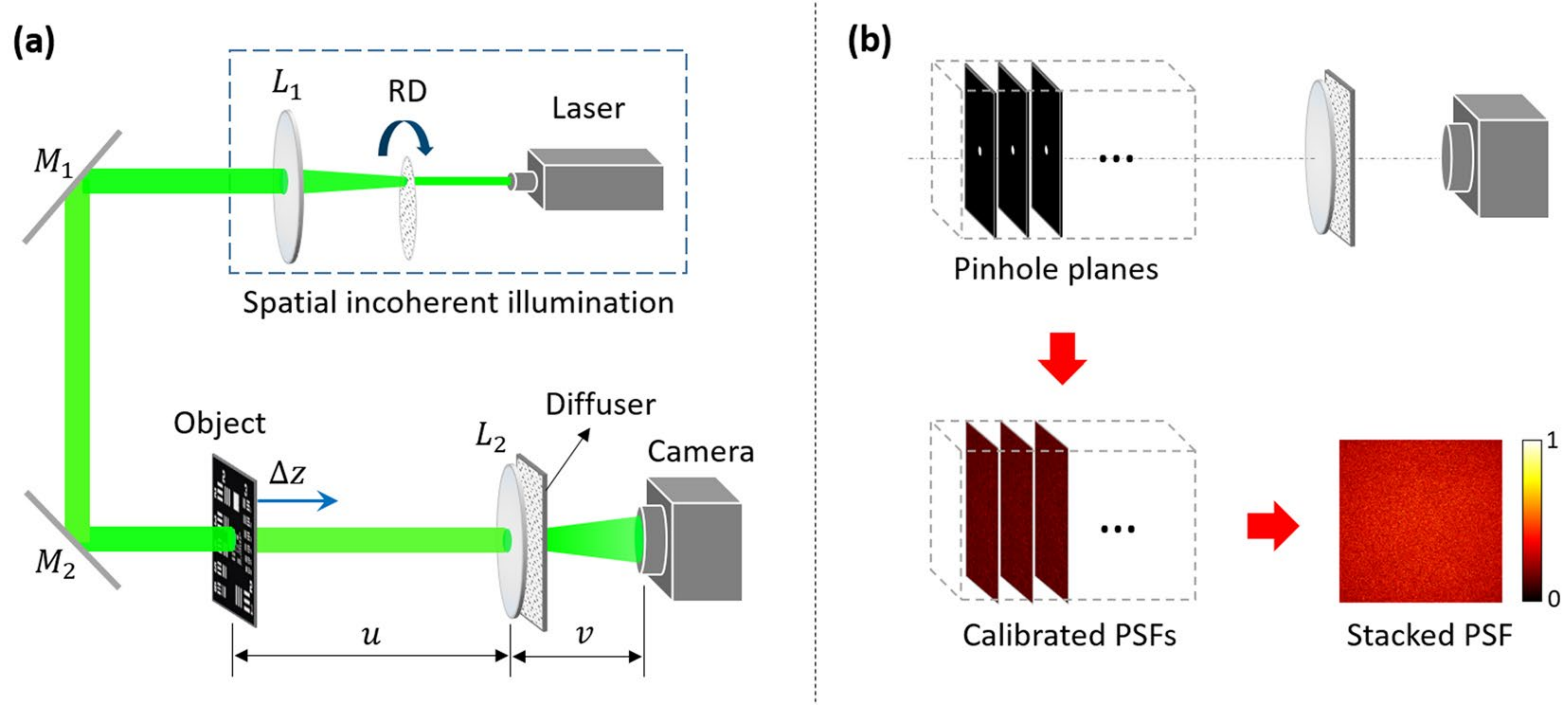
Experimental set-up and PSF calibration. (**a**) Schematic of optical experimental setup. (**b**) The calibrated PSFs in the measurement range and the stacked PSF by superimposing these calibrated PSFs.

We carried out experiments to demonstrate extended DOF imaging with a scattering diffuser in both a single lens system and a microscopic system. The experimental setup of single lens system is presented in Fig. 2(a). The spatially incoherent illumination was composed of a laser (*λ* = 532 nm) and a rotated scattering diffuser. To attain a relatedly wide field of view (FOV), a thin ground glass (DG20-220-MD, Thorlabs) was selected as the scattering diffuser element in the experiments. The camera was a high dynamic range CMOS (PCO edge 5.5, 2160 × 2160 pixels with a pixel size of 6.5 × 6.5 μm). *L*_1_ and *L*_2_ denote the collimating lens and the imaging lens, respectively. The focal length of the imaging lens *L*_2_ was *f* = 15 cm. *u* and *v* denote the object distance and image distance, respectively. When the object and camera were placed at the distances *u* = 60 cm and *v* = 20 cm on the opposite sides of the imaging lens *L*_2_, the Gaussian thin lens relation $1/f=1/u+1/v$ was followed. For the calibration, the diameter of the pinhole should be appropriately chosen by weighing the resolution and signal to noise ratio (SNR). The oversized pinhole will decrease the resolution of reconstructed images, whereas a pinhole with a smaller diameter can improve resolution but it will suffer from low SNR. Here a pinhole with a diameter of 50 μm was chosen and orderly placed at 20 axial positions, with the spacing interval of 1 cm in the whole measurement range of 20 cm. As presented in Fig. 2(b), the camera recorded the corresponding speckle patterns for each position. The obtained PSFs were then superimposed for generation of a stacked PSF.

**Figure 3:**
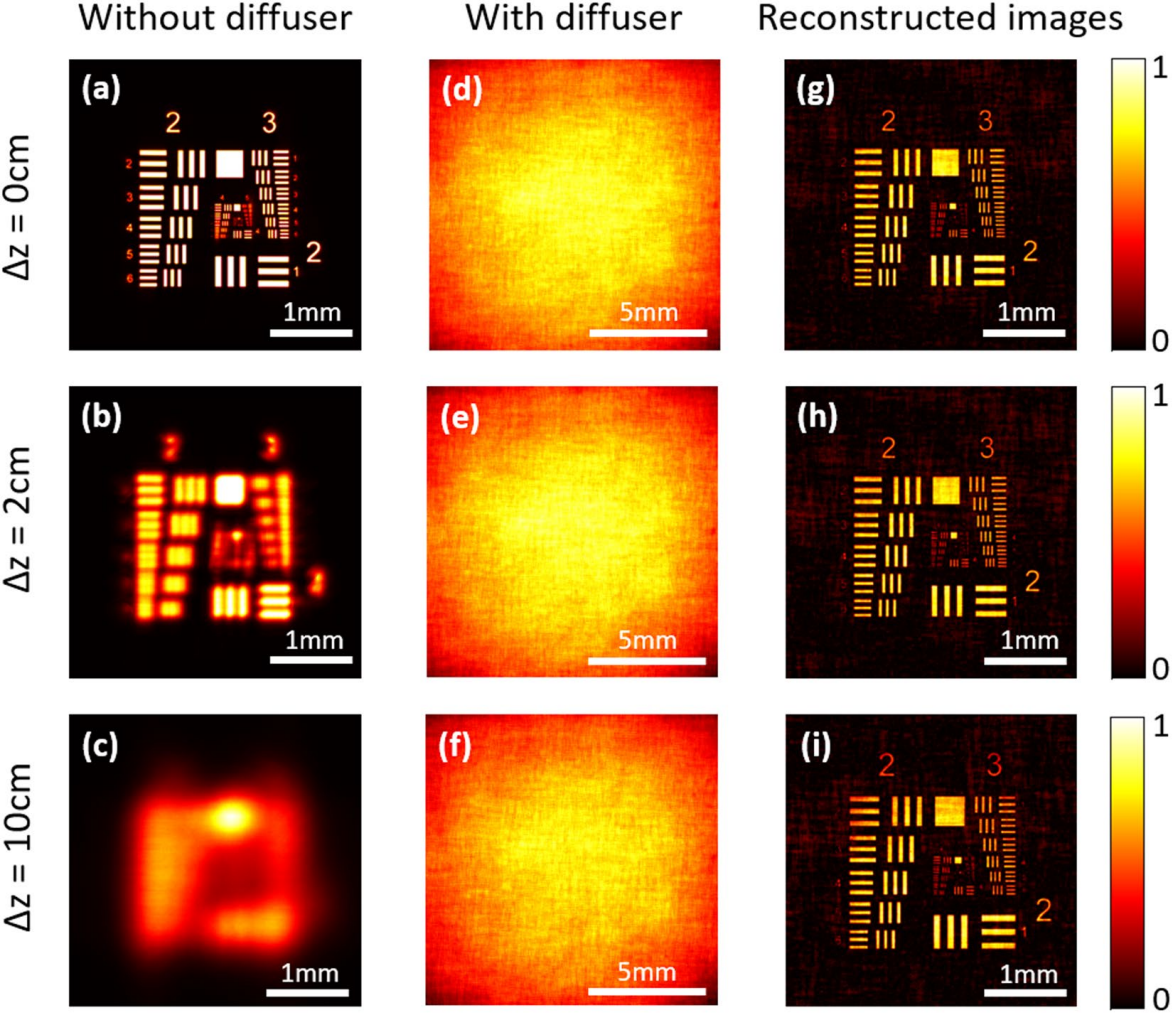
Imaging a planar object located at different axial position. (**a**–**c**) Conventional imaging of a planar object placed at various axial position. (**d**–**f**) The raw camera images obtained with a modified imaging system with a scattering diffuser. (**g**–**i**) The reconstructed images by deconvolution.

In the first experiment, a planar object (USAF resolution target, Thorlabs R3L3S1N), located at various distances from the imaging lens, was imaged. Figure 3(a–c) show the recorded images without scattering diffuser. When placed at the focus plane (∆*z* = 0 cm), the object was clearly imaged [see Fig. 3(a)]. When moved (∆*z* = 2 cm) close to the lens along the optical axis, it had blurred image [see Fig. 3(b)]. Further extending the defocused distance (∆*z* = 10 cm), the object could not be identified [see Fig. 3(c)]. After placing the scattering diffuser behind the lens and keeping other system parameters unchanged, the recorded pattern appeared heavily scrambled [see Fig. 3(d–f)]. However, sharp images were reconstructed by using the deconvolution with the stacked PSF [see Fig. 3(g–i)]. Notably, the resolution in reconstructed images with diffuser is limited the size of pinhole, it could be improved by using a smaller pinhole and a powerful light source. Meanwhile, scattering light intensity with diffuser led to the contrast reduction in the reconstructed images, but thus do reduces the dynamic range requirement of the detector. In addition, it should be noted that the PSFs of the different areas on the object are highly correlated with each other but not exactly the same. Therefore, the speckle pattern is just approximately equal to the convolution of the object with the intensity PSF, which resulting in the background noise in the reconstructed images. Furthermore, the proposed method took a relatively long time (more than 2 seconds) for recording a scattered PSF, which may introduce some unavoidable environmental noise. These noise influenced the deconvolution process and finally appeared on the reconstructed image as the type of background noise.

**Figure 4:**
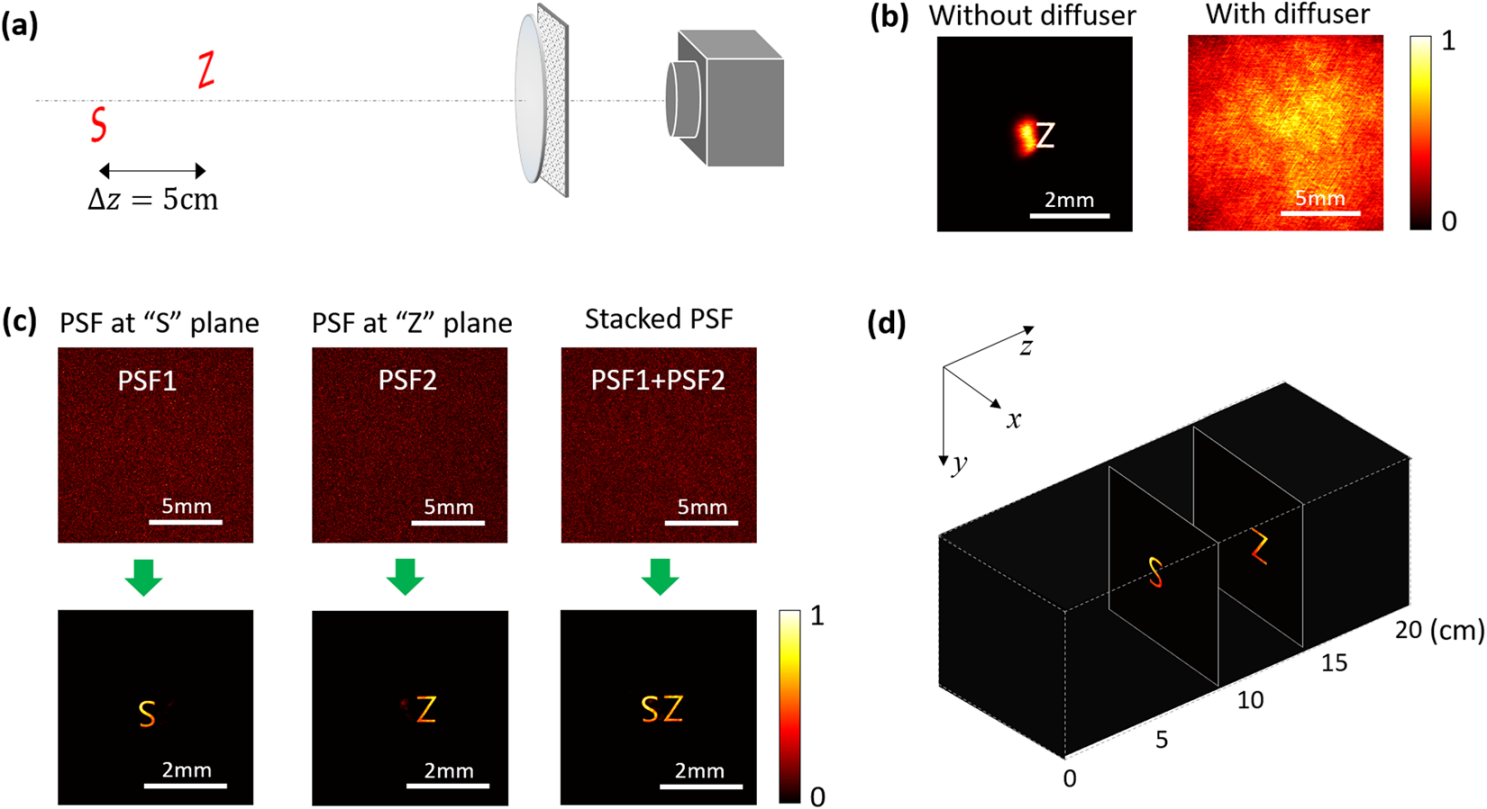
Imaging two objects at different positions along the optical axis. (**a**) Schematic of experimental setup. (**b**) Images obtained with a conventional imaging system without and with diffuser. (**c**) The recorded PSFs and the reconstructed images. **(d)** The reconstructed results in 3D coordinates.

In the second experiment, a three-dimensional scene consisting of two objects located at different positions along the optical axis was imaged. The experimental setup is depicted in Fig. 4(a). The imaged objects are two lithography masks (letters “S” and “Z” with 1 mm height), with the distance along the optical axis of ∆z = 5 cm. Figure 4(b) shows the raw recorded patterns without scattering diffuser and with scattering diffuser. In the conventional configuration (without scattering diffuser), the two objects could not be imaged clearly and sharply simultaneously. For instance, when the letter “Z” appeared sharp in the image, the letter “S” became blurred. With scattering diffuser, the recorded pattern became random. Figure 4(c) shows the PSFs and the corresponding reconstructed images. Both letters could be retrieved with the corresponding PSF. More importantly, they were recovered at the same time with the stacked PSF. Furthermore, as the 2D images of the objects located at different axial positions were retrieved by the PSF at corresponding planes, 3D imaging could be achieved slice by slice [see Fig. 4(d)].

**Figure 5:**
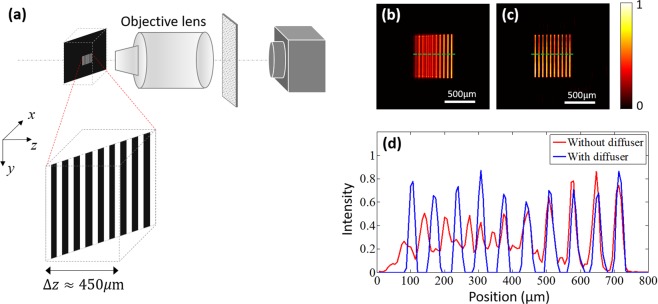
Imaging a depth extended object in a microscopic imaging system. (**a**) Schematic of experimental setup, (**b**) Conventional microscope imaging of a depth extended object. (**c**) The reconstructed image obtained with the proposed method. (**d**) The cross-sectional plots of the two images along the green lines.

In addition, the extended DOF in a microscopic imaging system was demonstrated as well. Figure 5(a) depicts the experimental configuration. An objective lens (4×/0.10NA) was used to produce a magnified image of the specimen. A pinhole with a diameter of 10 μm was chosen here and orderly placed at 50 axial positions, with the spacing interval of 10 μm in the whole measurement range of 500 μm. The camera exposure time was 8 s under the same power light source. A lithographic mask (10 vertical bars tilted in front of the objective lens. As shown in Fig. 5(a), the axial distance between the lines is around 450 μm. The DOF for a microscope objective^36^ was given by $DOF\approx \lambda n/(N{A}^{2})$, where *λ* is the wavelength, *n* is the index of refraction of the medium between the sample and objective, and NA is the objective’s numerical aperture. For this objective, the DOF was only around 100 μm. Figure 5(b) shows the images in a conventional microscopic imaging system (without scattering diffuser). The left part of image appeared blurred due to the defocusing. Figure 5(c) shows the reconstructed image with scattering diffuser. Features in all 10 vertical bars were resolved including some of the sharp edges. To better visualize the DOF extension, we show a line plot across the conventional image and the reconstructed image in Fig. 5(d). The DOF extension in microscopic imaging means that the working distance in microscopy could be enlarged, allowing more flexibility. The proposed method can be applied with higher NA objective lens under the diffraction-limit, but a smaller size pinhole and a higher power light source are needed to obtain a more accurate PSF for calibration thus allow for more reliable image reconstruction.

One of the feature of the proposed method is that there is no need to position the scattering diffuser accurately and just keep it unchanged during PSFs recording and speckle pattern measurement. Furthermore, the proposed method was demonstrated only in a transmission model and the imaging system was illuminated by a spatial incoherent light. The reflection model and the white light illumination need to be considered in the further research.

## Conclusion

In summary, we experimentally demonstrated a simple but effective method to extend the DOF of an imaging system. By introducing a thin scattering diffuser (such as a common ground glass) into the spatial incoherent imaging system, one can acquire the images with a larger DOF. Theoretical analysis was carried out by using the model based on the similarity between the optical transfer function and the ambiguity function. The statistical properties of the defocused OTF were analyzed by numerical simulations. Owing to the random scattering properties of the scattering diffuser, more high-frequency components that were lost with defocusing in conventional imaging could be collected. Although the detected image is a speckle pattern, the clear image could be easily recovered by using deconvolution with the calibrated PSF. The proposed approach does not require to design and fabricate special phase masks. Its effectiveness and robustness for extending the DOF were experimentally verified in both single-lens and microscopic imaging systems.
